# The Importance of Saccular Function to Motor Development in Children with Hearing Impairments

**DOI:** 10.1155/2009/972565

**Published:** 2010-01-27

**Authors:** Mary S. Shall

**Affiliations:** Department of Physical Therapy, Virginia Commonwealth University, P.O. Box 980224, Richmond, VA 23298-0224, USA

## Abstract

Children with hearing deficits frequently have delayed motor development. The purpose of this study was to evaluate saccular function in children with hearing impairments using the Vestibular Evoked Myogenic Potential (VEMP). The impact of the saccular hypofunction on the timely maturation of normal balance strategies was examined using the Movement Assessment Battery for Children (Movement ABC). Thirty-three children with bilateral severe/profound hearing impairment between 4 and 7 years of age were recruited from a three-state area. Approximately half of the sample had one or bilateral cochlear implants, one used bilateral hearing aids, and the rest used no amplification. Parents reported whether the hearing impairment was diagnosed within the first year or after 2 years of age. No VEMP was evoked in two thirds of the hearing impaired (HI) children in response to the bone-conducted stimulus. Children who were reportedly hearing impaired since birth had significantly poorer scores when tested with the Movement ABC.

## 1. Introduction

Most newborn infants are screened for hearing impairment prior to leaving the hospital. As a result, most parents are prepared for the choices of communication needs during this most important period of development. On the other hand, little attention is paid to the performance of the vestibular sensory system, whose receptors are located in the same area as the cochlear receptors. The vestibular nuclei provide a consistent input to the postural muscle motoneurons particularly during initial postnatal development [1]. The lateral vestibulospinal tract affects the vestibulospinal reflex by synapsing directly with motoneurons innervating extensor muscles and indirectly through spinal cord interneurons [2]. Obviously, the development of righting and locomotor skills should parallel sensory system development and motivation. This sensorimotor development has been more extensively studied in nonhuman mammals as they develop motor skills during the first four months [3, 4]. The ferret is born with sensory systems that are more immature than that of the rat or cat. The eyes open at about postnatal day 28 (P28) [5] and hearing becomes functional at about P32 [6]. The vestibular receptors mature at about the same rate as the auditory system; the maculae of the saccule and utricle mature first (~P10) and the cristae of the semicircular canals develop a little more slowly to reach the adult form at about 24 days after birth [7], and the ferrets begin walking at about P24. Neonatal removal of the vestibular labyrinths in ferrets [8] and rats [9] has resulted in changes in muscle physiology and muscle fiber type, persistent unstable standing balance, and reduced velocity of locomotion. Animals that undergo labyrinthectomies as a juvenile or an adult may have some loss of balance reactions but adapt quickly and do not change the anatomy and physiology of the muscles. 

In contrast, vestibular receptors have been seen under the microscope in 7–9-week-old human embryos [10], but the vestibular reflexes develop a little more slowly in humans as the sensory systems continue to mature and integrate. Shumway-Cook and Woollacott proposed that, normally, children undergo transition to more adult strategies of posture control between the ages of 4 and 6 [11]. Rine et al. [12] examined the gross motor development among young children with bilateral moderate to profound sensorineural hearing impairment (HI). Delayed gross motor development was evident in these children and a majority received “vestibular hypofunction scores” using the Southern California Postrotary Nystagmus Test (PRN), Ayres (1983), Western Psychological Services. The PRN test measures the responsiveness of the lateral semicircular canal by assessing the vestibular ocular reflex which occurs in response to rotation. It is designed for children of normal intelligence between the ages of 5 and 9. In comparison, the Vestibular Evoked Myogenic Potential (VEMP) evaluates saccular function by stimulating those receptors and recording the time delay to the evoked EMG recorded from the sternocleidomastoid muscle [13]. 

The primary purpose of this study is the identification of functioning vestibular saccule receptors among children with hearing impairments and correlation of saccular deficits with fine and gross motor developments. A secondary aim was the comparison of motor skills in a group of children with hearing impairment from birth with a group of children that lost their hearing after 2 years of age.

## 2. Methods

Thirty-three children (20 males, 13 females) with bilateral severe/profound sensorineural hearing impairments (HIs) (loss greater than 71 dB) were recruited as a sample of convenience through parent support groups such as the Virginia Chapter of the AG Bell Association, the Speech-Language-Hearing Association of Virginia, and the North Carolina Triad Hitch-Up. The cause of hearing loss was unknown in the majority of the cases. Two mothers said they had a cytomegalovirus infection during pregnancy. Two other children had tested positively for the connexin 26 gene but most had not been tested for genetic markers. All children were between the ages of 4 and 7 years (mean = 66.1 ± 12.3 months). There were 3 other children that refused the test either prior to any testing or during the test; 2 were 4 years old and 1 was 5 years old. One child had bilateral hearing aids. Nineteen of the children had cochlear implants for at least one year prior to testing (range = 1–5 years). Five of the nineteen children only had a cochlear implant in one ear. Parents or guardians completed forms providing a brief health history on the child and consented to perform the evaluation. Most presented audiological documentation. Twenty-four of the children had hearing impairment since birth but no other obvious neurologic damage. 

The Vestibular Evoked Myogenic Potential (VEMP) was used to evaluate saccular function. The child lay supine on a mat on the floor with the investigator in a separate quiet room and frequently a parent was in the immediate proximity. The skin over the sternocleidomastoid (SCM) muscles and sternum were cleaned with an alcohol wipe. A surface bar electrode (2 concave Ag/AgCl electrodes separated by 3 cm and coated with conductivity gel) was placed on the SCM muscle (active on the muscle belly, reference on the tendon), on the same side as the stimulus, to record the electromyographic potential (EMG). A ground electrode was placed on the sternum. The EMG was amplified and bandpass filtered (8 Hz–1.6 kHz). A 10-ohm, 15 pin FA Bone Vibrator transducer (Cadwell Laboratories, Inc., Kennewick, WA, USA, with impedence matching adaptor box) was held against the mastoid process while the child flexed his or her head against gravity to activate the SCM while looking at a picture book or playing with a toy. Preliminary trials showed that the children were reluctant to use the earphone/AC stimulation (maybe it was associated with hearing tests) though they were willing to use the bone conducted stimulus. One hundred *μ*s clicks were generated by the Cadwell Wedge (Kennewick, WA) at 95 dB, 16 Hz. 95 dB is commonly used in the literature for air- and bone-conducted VEMP though there have been reports of a less intense stimulus (e.g., 50 dB) [14]. A consistent stimulus that was well tolerated by all participants was used in this case. The baseline EMG activity of the SCM was visualized and then 200 tracings of the evoked potential were averaged. The left side was always tested first and the procedure was repeated on the right side. The latency and amplitude of the potential was visualized and quantified using Cadwell Sierra XP software (Kennewick, WA). Based on normative data (using air-conducted VEMP) on young children [15], a normal p1 latency was in the range of 8.3–14.4 seconds and the VEMP at least 20 mV amplitude. Todd et al. [16] have shown a slightly wider range of VEMP latencies (7.5–13.9) for bone-conducted stimulation than air-conducted stimulation in adults. 

Each child was evaluated using the Movement Assessment Battery for Children (Movement ABC) (Psychological Corp., London, England, 1992) to assess their proficiency in areas of manual dexterity, ball skills, and static and dynamic balances (Table 1). If the child wore cochlear implants, these were allowed during the motor testing (but not during VEMP testing). This standardized test is designed for use with children aged four to twelve years. The raw scores are converted into scaled scores in order to ascertain where the child's performance lies in relation to the standardized sample. The sum of the three subtests provides a total impairment score. Higher scores indicate greater impairment. The test has excellent reliability (*r* = 0.96) and validity in normal children and children with disabilities and from populations in Europe and North America [17–19]. 

Student's *t*-tests were used to compare the groups of subjects with a limit of *P* < .05 indicating significance.

## 3. Results

**Figure 1:**
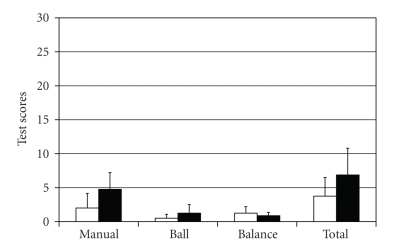
Plot of Movement ABC scores by test subset in children with VEMP present on both sides. White columns = children with normal hearing; Dark columns = HI children.

A VEMP was identified on both sides in only 4 of the HI children (3 of these children were identified as HI at birth). Four children with normal hearing of the same age as the four HI children with normal VEMP were recruited for comparison. There were no significant differences between these two groups in the total or subscores on the Movement ABC (Figure 1) though the HI children tended to have greater impairment on the manual subtest (*P* < .07) and ball subtest (*P* < .2) using a paired *t*-test. 

All of the figures are scaled to the same number to allow easy comparison. It should be noted that power analysis indicated that to reject the null hypothesis correctly at the .05 level, a sample size of 14 was needed. So caution should be taken in the interpretation of this limited number of subjects though the scores indicate that all of the children in Figure 1 are functioning similarly to the standardized group. 

**Figure 2:**
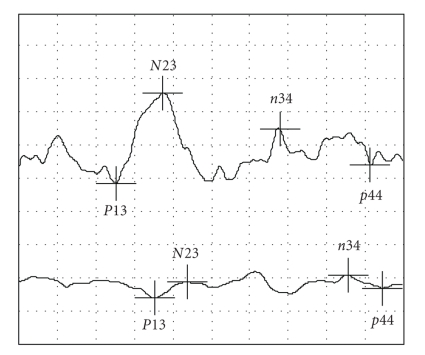
Asymmetrical VEMP recorded from a 5-year-old HI subject. Left side is above (P13 = 11.7 ms) and right side is below. Horizontal square = 5 ms and vertical square = 20 *μ*v.

**Figure 3:**
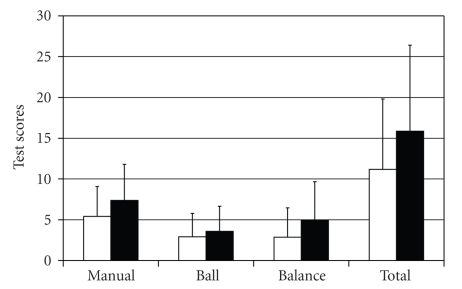
Plot of Movement ABC scores by test subset in HI children. White columns = HI children with a VEMP response on one or both sides (*N* = 7); Dark columns = HI children with no VEMP (*N* = 11).

Seven children demonstrated a VEMP on only one side (Figure 2) as measured by the action potential amplitude >20 *μ*v and latency (mean = 10.3 ± 1.5 ms). There was a tendency (*P *< .9–.26) for HI children with one or both sides intact (*n* = 11) to perform better on the Movement ABC than HI children with no VEMP (*n* = 23) (Figure 3). Of the HI children with an asymmetrical response, one child had a right cochlear implant and VEMP only on the left side, while the rest of the children had bilateral cochlear implants (*n* = 2) or no implants (*n* = 4). 

**Figure 4:**
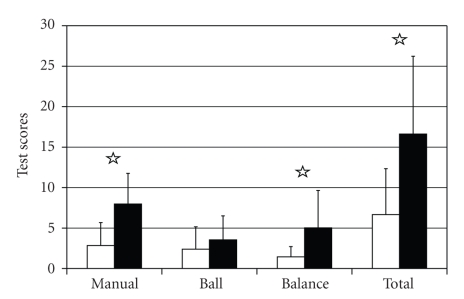
Plot of Movement ABC scores by test subset in children with 1 or no VEMP present. White columns = HI children who lost hearing later (*N* = 9); Dark columns = HI children who lost hearing neonatally (*N* = 24). (Star = *P *< .01.)

Twenty-four children were HI since birth or within 6 months (according to the parents); nine children developed HI after the age of two years (7 at about 24 months, 2 at about 36 months). Two years was used as the natural dividing point between groups. The children with HI since birth had significantly higher scores (more developmental delay) (*P *< .01) on the manual and balance subtests and the total Movement ABC (Figure 4). Two of the children in each group retained their saccular receptors bilaterally as shown by VEMP. 

No significant difference was seen in the Movement ABC scores between the HI children with and without cochlear implants (mean of the total ABC scores = 12.6 ± 9.6 and 15.5 ± 9.9, resp.).

## 4. Discussion

The results of this study are important to show that many but not all hearing impaired children have saccular deficit (s). A more strategic finding is the importance of the timing of the onset of the hearing impairment and probably saccular loss to the development of balance strategies. 

The early postnatal period is critical for the vestibular system in experimental models such as the ferret, so that it impacts neuromuscular development of postural muscles and the development of balance strategies [8]. Human studies have focused more on motor development in the deaf population as a uniform population [12] or the effect of cochlear implants. There have been several studies of motor development of HI children with mixed results [20–24]. Some studies [20, 21] that have included HI children younger than 7 years old also include children up to 17 years of age. Rine et al. [12] noted that HI children did have delayed motor development which seemed to decelerate annually. They related this delay to “vestibular hypofunction” and supported the need for vestibular testing and repeated motor development testing during preschool and elementary school. It is unknown wether all of the vestibular receptors are lost at the same time as the cochlear receptors but the parents in this study report that the hearing loss was identified in many of the children neonatally, implying the need for very early identification of children with vestibular deficits. The current study found a significant difference in the motor development between the children with a profound hearing loss at birth and those that reportedly developed HI later with those with presumably congenital onset hearing loss demonstrating poorer performance. It is possible that vestibular receptors are more important during the initial six months to a year when the infant is learning to sit and stand. Normally children are walking by two years of age, albeit with a wide based gait. It is likely that HI children, who also have a vestibular deficit (at least a loss of saccular function), learn to depend on their vision and joint and skin sensation but it is hypothesized that the developmental milestones such as walking would be delayed without the vestibulospinal reflexes. 

Nineteen of the HI children in this sample also had cochlear implants. Some of the parents reported that there were 1-2 days post implantation when the children seemed to be a little more “clumsy,” after which the children quickly returned to their presurgical motor coordination. This brief change was not measured, and at the time of this study, the children with and without cochlear implants were not significantly different in their performance of the Movement ABC. The VEMP was normal on at least one side in six (of 19) of the HI children with cochlear implants. This would seem to indicate that the cochlear implant does not have a long-term effect on balance and motor control. Cushing et al. [25] studied 40 children with profound SNHL and unilateral cochlear implants. The children ranged in age from 3 to 19 years. Saccular function (tested by air-conducted VEMP) was found in 40% of their subjects but was not correlated with balance ability when tested by the balance subset of the Bruininks-Oseretsky Test of Motor Proficiency II. In contrast, horizontal canal function correlated well with the balance score which implies that angular motion and vestibulo-ocular interaction are very important to balance. The saccule detects more linear motion so the correlation of saccule hypofunction with both delayed fine motor skills and balance skills leads one to conclude that the saccule may impact both the vestibulospinal and vestibulo-ocular reflexes though its differential function remains unclear. Only the VEMP was used in this study and, therefore, only the saccule receptors were studied. There are other vestibular evaluation measures such as videonystagmography (VNG), Rotary Chair, bithermal caloric irrigation, and Computerized Dynamic Posturography which evaluate other vestibular receptors. Hence, it is possible that another vestibular receptor may have impacted performance. As with all motor disorders, it is important to take into consideration that vision, somatosensory, vestibular, and sensations are integrated to provide optimal performance of motor control. 

A limitation of the convenience sample used in this study is that parents who may have been concerned about balance dysfunction may have volunteered their children for this study. All of these parents were eager to maximize the potential development of their children.

## 5. Conclusion

The onset of hearing impairment (congenital versus delayed, potentially progressive) has an impact on balance and manual skills and overall motor development. How this difference relates to the development or impairment of the vestibular receptors (in the case of the current study—saccular function) is unclear and needs further study. What is happening at the level of the cochlea may parallel what is happening at the level of the vestibular end organs. The absence of VEMP bilaterally may predict poor performance on balance and manual dexterity- related tasks although a larger sample would be required to say that this is more than a trend. The VEMP has only recently been used for screening of babies [26–28]. Further research is necessary to correlate the timing of the loss of saccular receptors with motor development in children. Early intervention could potentially optimize postural control in children who are identified with vestibular hypofunction [29].

## Figures and Tables

**Table 1:** Skills quantified by the Movement ABC.

Skill categories	Tasks
Manual dexterity	Insert coins in a box (with preferred and nonpreferred hands)
	Threading beads
	Trace bicycle trail with pen

Ball skills	Catch bean bag
	Roll ball into goal

Static and dynamic balances	One-leg balance (preferred and non preferred legs)
	Jump over a cord at knee level
	Walking with heels raised

## References

[1] Christensson M, Garwicz M (2005). Time course of postnatal motor development in ferrets: ontogenetic and comparative perspectives. *Behavioural Brain Research*.

[2] Grillner S, Hongo T, Lund S (1970). The vestibulospinal tract. Effects on alpha-motoneurones in the lumbosacral spinal cord in the cat. *Experimental Brain Research*.

[3] Lelard T, Jamon M, Gasc J-P, Vidal P-P (2006). Postural development in rats. *Experimental Neurology*.

[4] Sechzer JA, Folstein SE, Geiger EH, Mervis RF, Meehan SM (1984). Development and maturation of postural reflexes in normal kittens. *Experimental Neurology*.

[5] Issa NP, Trachtenberg JT, Chapman B, Zahs KR, Stryker MP (1999). The critical period for ocular dominance plasticity in the Ferret’s visual cortex. *Journal of Neuroscience*.

[6] Moore DR (1982). Late onset of hearing in the ferret. *Brain Research*.

[7] Shall MS, Lanzino DJ, Kinirons SA (1999). Postnatal development of the ferret vestibular receptors. *Society for Neuroscience Abstracts*.

[8] Van Cleave S, Shall MS (2006). A critical period for the impact of vestibular sensation on ferret motor development. *Journal of Vestibular Research: Equilibrium and Orientation*.

[9] Ito J, Nakajima K, Mori S (1998). Postnatal changes in locomotor movements after labyrinthectomy in rats. *Neuroscience Research*.

[10] Sans A, Dechesne C (1985). Early development of vestibular receptors in human embryos: *an electron microscopic study*. *Acta Oto-Laryngologica*.

[11] Shumway-Cook A, Woollacott MH (1985). The growth of stability: postural control from a developmental perspective. *Journal of Motor Behavior*.

[12] Rine RM, Cornwall G, Gan K (2000). Evidence of progressive delay of motor development in children with sensorineural hearing loss and concurrent vestibular dysfunction. *Perceptual and Motor Skills*.

[13] Halmagyi GM, Curthoys IS (1999). Clinical testing of otolith function. *Annals of the New York Academy of Sciences*.

[14] Monobe H, Murofushi T (2004). Vestibular neuritis in a child with otitis media with effusion; clinical application of vestibular evoked myogenic potential by bone-conducted sound. *International Journal of Pediatric Otorhinolaryngology*.

[15] Kelsch TA, Schaefer LA, Esquivel CR (2006). Vestibular evoked myogenic potentials in young children: test parameters and normative data. *The Laryngoscope*.

[16] Todd NPM, Rosengren SM, Aw ST, Colebatch JG (2007). Ocular vestibular evoked myogenic potentials (OVEMPs) produced by air- and bone-conducted sound. *Clinical Neurophysiology*.

[17] Van Waelvelde H, De Weerdt W, De Cock P, Smits-Engelsman BCM (2004). Aspects of the validity of the movement assessment battery for children. *Human Movement Science*.

[18] Riggen KJ, Ulrich DA, Ozmun JC (1990). Reliability and concurrent validity of the test of motor impairment-Henderson revision. *Adapted Physical Activity Quarterly*.

[19] Schoemaker MM, Smits-Engelsman BCM, Jongmans MJ (2003). Psychometric properties of the Movement Assessment Battery for Children-Checklist as a screening instrument for children with a developmental co-ordination disorder. *British Journal of Educational Psychology*.

[20] Horn DL, Pisoni DB, Miyamoto RT (2006). Divergence of fine and gross motor skills in prelingually deaf children: implications for cochlear implantation. *The Laryngoscope*.

[21] Cushing SL, Chia R, James AL, Papsin BC, Gordon KA (2008). A test of static and dynamic balance function in children with cochlear implants: the vestibular olympics. *Archives of Otolaryngology—Head and Neck Surgery*.

[22] Suarez H, Angeli S, Suarez A, Rosales B, Carrera X, Alonso R (2007). Balance sensory organization in children with profound hearing loss and cochlear implants. *International Journal of Pediatric Otorhinolaryngology*.

[23] Horak FB, Shumway-Cook A, Crowe TK, Black FO (1988). Vestibular function and motor proficiency of children with impaired hearing, or with learning disability and motor impairments. *Developmental Medicine and Child Neurology*.

[24] Gayle GW, Pohlman RL (1990). Comparative study of the dynamic, static, and rotary balance of deaf and hearing children. *Perceptual and Motor Skills*.

[25] Cushing SL, Papain BC, Rutka JA, James AL, Gordon KA (2008). Evidence of vestibular and balance dysfunction in children with profound sensorineural hearing loss using cochlear implants. *The Laryngoscope*.

[26] Zagólski O (2007). Vestibular system in infants with hereditary nonsyndromic deafness. *Otology & Neurotology*.

[27] Sheykholesami K, Kaga K, Megerian CA, Arnold JE (2005). Vestibular-evoked myogenic potentials in infancy and early childhood. *The Laryngoscope*.

[28] Shinjo Y, Jin Y, Kaga K (2007). Assessment of vestibular function of infants and children with congenital and acquired deafness using the ice-water caloric test, rotational chair test and vestibular-evoked myogenic potential recording. *Acta Oto-Laryngologica*.

[29] Rine RM, Braswell J, Fisher D, Joyce K, Kalar K, Shaffer M (2004). Improvement of motor development and postural control following intervention in children with sensorineural hearing loss and vestibular impairment. *International Journal of Pediatric Otorhinolaryngology*.

